# Linking Behavior, Co-infection Patterns, and Viral Infection Risk With the Whole Gastrointestinal Helminth Community Structure in *Mastomys natalensis*

**DOI:** 10.3389/fvets.2021.669058

**Published:** 2021-08-17

**Authors:** Bram Vanden Broecke, Lisse Bernaerts, Alexis Ribas, Vincent Sluydts, Ladslaus Mnyone, Erik Matthysen, Herwig Leirs

**Affiliations:** ^1^Evolutionary Ecology Group, Department of Biology, University of Antwerp, Antwerp, Belgium; ^2^Parasitology Section, Department of Biology, Healthcare and Environment, Faculty of Pharmacy and Food Science, IRBio (Research Institute of Biodiversity), University of Barcelona, Barcelona, Spain; ^3^Pest Management Center, Sokoine University of Agriculture, Morogoro, Tanzania

**Keywords:** *Mastomys natalensis*, helminths, endoparasites, exploration behavior, animal personality, co-infection, infection heterogeneity, morogoro arenavirus

## Abstract

Infection probability, load, and community structure of helminths varies strongly between and within animal populations. This can be ascribed to environmental stochasticity or due to individual characteristics of the host such as their age or sex. Other, but understudied, factors are the hosts' behavior and co-infection patterns. In this study, we used the multimammate mouse (*Mastomys natalensis*) as a model system to investigate how the hosts' sex, age, exploration behavior, and viral infection history affects their infection risk, parasitic load, and community structure of gastrointestinal helminths. We hypothesized that the hosts' exploration behavior would play a key role in the risk for infection by different gastrointestinal helminths, whereby highly explorative individuals would have a higher infection risk leading to a wider diversity of helminths and a larger load compared to less explorative individuals. Fieldwork was performed in Morogoro, Tanzania, where we trapped a total of 214 individual mice. Their exploratory behavior was characterized using a hole-board test after which we collected the helminths inside their gastrointestinal tract. During our study, we found helminths belonging to eight different genera: *Hymenolepis* sp., *Protospirura muricola, Syphacia* sp., *Trichuris mastomysi, Gongylonema* sp., *Pterygodermatites* sp., *Raillietina* sp., and *Inermicapsifer* sp. and one family: *Trichostrongylidae*. Hierarchical modeling of species communities (HMSC) was used to investigate the effect of the different host-related factors on the infection probability, parasite load, and community structure of these helminths. Our results show that species richness was higher in adults and in females compared to juveniles and males, respectively. Contrary to our expectations, we found that less explorative individuals had higher infection probability with different helminths resulting in a higher diversity, which could be due to a higher exposure rate to these helminths and/or behavioral modification due to the infection.

## Introduction

Helminths (Nematoda, Cestoda, and Trematoda), or parasitic worms, are a well-studied and widespread parasitic group with a large impact on human health. In low to middle income countries, more than one billion people are infected with soil-transmitted helminths alone (1, 2). In addition, they have large economic consequences as well, for example in the livestock industry (3, 4). Most animal species, however, face a wide diversity of helminths at the same time. The community structure of these helminths can vary between populations, due to differences in environmental conditions such as temperature, rainfall, and urbanization (5–8) as well as within populations, where some individuals are infected with a wider diversity of helminths than others. Indeed, multiple parasitic infections (i.e., co-infections) within a single host occurs very frequently in the wild and may have a large effect on both the infection heterogeneity among individuals as well as on the parasitic communities within a host (9–12).

Such co-infection patterns within a single host can arise through common risk factors, such as environmental conditions, or via the host's behavior and space use (11). Indeed, the hosts' behavior has been found to affect both their exposure to different parasites as well as their susceptibility to become infected by them (13–15). But this may vary among individuals since not all individuals behave in a similar way. During the last two decades it has been shown that there are consistent differences in behavior among individuals through time and/or contexts within a population (16, 17). This phenomenon is commonly referred to as animal personality and has been found in a wide variety of taxa (18, 19) affecting survival (20, 21), and reproductive success (22, 23). It has also been hypothesized to influence parasitic infection heterogeneity, where bolder and more explorative individuals are thought to have a higher infection risk compared to shyer, less explorative individuals (24). This is based on the assumption that these bolder, and more explorative, individuals are more active and take more risks in the wild which increases their probability to come into contact with an infected individual or environment (24, 25). Indeed, this has been confirmed in several chipmunk species where more explorative and bolder chipmunks had a higher ectoparasite [*Tamias minimus* (26) and *Tamias sibiricus* (25)] and endoparasite load [*Tamias striatus* (27, 28)]. Similar results have been found in great tits (*Parus major*), where avian malaria infection occurred more frequently in highly explorative individuals (29).

However, this relationship between the host's personality and parasitic infection probability might be more complex than generally assumed. Personality in eastern gray squirrels (*Sciurus carolinensis*), for instance, was associated with infection probability but not with infection intensity of the helminth *Strongyloides robustus* (30). Another example is the European green lizard (*Lacerta viridis*) where adult males without ticks (*Ixodes* spp.) were more explorative than males that were parasitized (31). These contrasting results could be the effect of different transmission strategies of the parasite (32), parasitic manipulation of the host's behavior (15, 24, 33) but also due interactions among the different parasites inside the host. Such interactions can, on the one hand, be antagonistic which means that the presence of one parasite could inhibit subsequent infections, due to resource competition (11, 12). Budischak et al. (34) has shown, for example, that bloodsucking helminths compete with malaria for red blood cells and that malaria densities inside the host increased significantly in dewormed populations. On the other hand, parasites may also have a more synergetic interaction among them where the presence of one parasite facilitates subsequent infections due to, for instance, reducing the host's immunity (11). Wood mice (*Apodemus sylvaticus*), for instance, infected with the nematode *Heligmosomoides polygyrus* were more likely to be infected with other helminth species, potentially due to the immunodepressive effect of *H. polygyrus* (35, 36). But this may also affect other parasites besides helminths such as bacteria (37). Ezenwa et al. (38), for instance, has shown that nematode-induced immune suppression facilitated the invasion of bovine tuberculosis in free ranging African buffalos (*Syncerus caffer*). But helminths might also affect the host's immunity against viruses, which may have important consequences on the viral transmission dynamics within a populations (37). Indeed, several experiments have revealed that both viral loads and clearance time was higher in helminth co-infected mice compared to those without helminths (37, 39, 40). Nonetheless, no study has (as far as we are aware of) investigated how both the hosts' personality and co-infection patterns jointly shape the variation in parasite communities of helminth and viral infection risk among-individuals in a wild mammal species.

In this study, we used the multimammate mouse (*Mastomys natalensis*) as a model system to investigate how the hosts' age, sex, and behavior affect the individuals' infection risk, parasite load, and community structure of the different gastrointestinal helminths and how this, in turn, influences their viral infection history with a common arenavirus (Morogoro virus, MORV). *Mastomys natalensis* is the most common indigenous rodent in sub-Saharan African and host for a wide variety of parasites, such as several arenaviruses (41–45), plague bacteria (46), cutaneous leishmaniasis (47) as well as different ecto- (48, 49) and endoparasites which have been studied extensively over the years (50–57). They are therefore an excellent model system to study the interaction between behavior and parasitic communities, especially since it has been found that there are consistent differences in behavior among individuals. Indeed, *M. natalensis* has been shown to express two separate personality traits: exploration and stress-sensitivity, which means that within a population, some individuals are consistently more explorative, or stress-sensitive than others (58, 59). These differences in personality have been found to be associated with viral infection probabilities, where less explorative individuals had a higher probability of getting infected with the MORV compared to highly explorative individuals (59). Co-infection patterns with this virus and associated behaviors could therefore potentially influence other parasites as well.

We therefore hypothesize that the host's exploration behavior has a large effect on the gastrointestinal helminth community structure and on their co-infection patterns in *M. natalensis*. Indeed, exploration has been found to positively affect helminth infection risk in other species (27, 60). Potentially because highly explorative individuals have a higher energetic demand (61, 62) compared to less explorative individuals and therefore need to spent more time foraging which increases their likelihood to encounter infected intermediate hosts and/or contaminated environments (17). These risks are potentially more pronounced in *M. natalensis* since their home ranges overlap greatly during periods of high resource availability in combination with low levels of territoriality (63) and their generalist diet (64). We therefore predict that highly explorative individuals are infected with a wider diversity of helminths and with a higher load (total number of helminths) compared to less explorative individuals, which may be enhanced by potential synergistic interactions among the different parasites (11). Additionally, we predict that helminth species richness is higher in males than females, due to the immunosuppressive effect of testosterone (65, 66) and that adults have a higher species richness than juveniles because they have had more opportunities to come into contact with different helminths (67). Lastly, individuals with antibodies against MORV [and were thus previously infected with MORV (68)] have been found to have a lower survival probability compared to those without antibodies, even though pathogenicity of MORV is not severe (69, 70). This could be due to current unknown co-infection patterns with helminths which reduces the host's health and makes them consequently more susceptible to become infected with MORV. We therefore hypothesize that MORVab positive individuals are infected with a higher diversity of helminths as well compared to MORVab negative individuals.

## Materials and Methods

### Study Site and Trapping

The fieldwork was conducted on the campus and university farms of the Sokoine University of Agriculture (SUA) in Morogoro, Tanzania, from July until September 2019. This period coincides with the breeding season of *M. natalensis*, which starts in March–May, in which the population size increases rapidly reaching a peak in October (71, 72). Rodents were trapped on 11 different sites in both maize fields and in fallow lands (Supplementary Figure 1). In each trapping site, we placed 80–200 Sherman LFA live traps (Sherman Live Trap Co., Tallahassee, FL) in lines of 10 with a distance of 10 m from each other, with a mix of peanut butter and maize flour as bait. Traps were placed in the late afternoon and were checked in the early morning (see Supplementary Table 1 for a more detailed description of the trapping effort per site). The trapped rodents were then brought to the Pest Management Center at SUA by car where we measured their behavior before they were euthanized and dissected.

### Behavioral Tests

The individuals' behavior was measured just once immediately after they arrived at the Pest Management Center around 10 a.m., using a hole-board test (59). This setup is a derivative of the open field test with blind holes in the floor (73). The box (75 × 55 × 90 cm; L × W × H, respectively) was constructed out of strong white plastic with six blind holes in the bottom (Ø: 3.5 cm; depth: 6 cm) each spaced 19 cm apart from each other. The box was closed off with a lid with a small hole for the infrared camera. Behavioral recordings started when the individual was inside the box and the lid was closed and lasted for 10 min. During this period, we measured five different behaviors: activity (the number of times an individual crossed one of the 12 squares), the number of times they sniffed a hole, number of head dips [when both eyes and ears disappear into one of the blind holes (73, 74)], the time they spent grooming, and the number of jumps. A more detailed description of the behavioral analysis can be found in Vanden Broecke et al. (59). The box was cleaned with 70% ethanol to remove animal scent and dirt. We ran two test simultaneously which reduced the time that was needed to test all the individuals on a single day which was on average 74 min (range = 1–221 min). Individuals were kept inside their traps at the Pest Management Center before being tested. A preliminary analysis has revealed that this had no effect on their behavior.

### Parasitological Examination

The individuals' body weight, sex, and reproductive status was recorded after the hole-board test, but before they were euthanized, following Leirs et al. (75). Blood samples were taken from the retro-orbital sinus when the animal was still alive and was preserved on pre-punched filter paper (Serobuvard, LDA 22, Zoopole). These blood samples were later analyzed at the University of Antwerp for MORV-specific IgG antibodies using immunofluorescence assay protocols described in Günther et al. (42) and Borremans (76).

The rodents were euthanized using a halothane overdose by placing them in a glass jar with cotton balls drenched in halothane for 40 min, followed by a cervical dislocation. We then removed the whole gastrointestinal tract, which we stored in a 50 ml tube with 100% ethanol for further analysis at the parasitology lab at the University of Barcelona (77). Here, the gastrointestinal tract was first placed in a petri dish filled with tap water in order to soften them. Afterwards, we separated the stomach, intestines, caecum, and colon from each other and were placed in separate petri dishes. Each of these organs was then carefully cut open and the content was checked under a stereomicroscope. The helminths were identified to genus or species level using morphological characteristics (54, 55).

All experimental procedures were approved by the University of Antwerp Ethical Committee for Animal Experimentation (2016-63), adhered to the EEC Council Directive 2010/63/EU, and followed the Animal Ethics guidelines of the Research Policy of the SUA.

### Statistical Analysis

We trapped 214 individuals (*N*_male adult_ = 54, *N*_male juvenile_ = 21, *N*_female adult_ = 114, *N*_female juvenile_ = 25) during our study period. We recorded the behavior of all these individuals, after which they were euthanized and dissected in order to investigate the helminths. A large proportion of the adult females (61.4%) were found to be pregnant after they were dissected.

### Behavioral Analysis

We followed the behavioral analysis of Vanden Broecke et al. (59) which allowed us to compare our results with the previous work on animal personality in *M. natalensis* (58, 59, 78, 79). We therefore ran a principal component analysis (PCA) on the five different behaviors (activity, hole sniffing, head dipping, jumping, and time spent grooming) measured in the hole-board test in order to reduce the number of variables (i.e., the five different behaviors). The Kaiser-Guttman criterion [eigenvalue >1 (80, 81)] was used to select the number of components to retain.

In order to use these components in the further analysis we needed to make sure that these behavioral components did not correlate with the different independent covariates (see below) during the HMSC modeling. We therefore created two linear mixed models (LMM) with the two first components from the PCA as response variables with a Gaussian error distribution. We added sex (male or female) and reproductive age (adult or juvenile) as well as their interaction as fixed effects. The area where the individual was trapped was added as a random effect. The residuals from these two models were used in further analysis as indices for the individuals behavior (25). These analyses were executed using the R software 4.03 (82) with the lme4 package [version 1.1-25 (83)].

### Parasitological Examination

We used Hierarchical Modeling of Species Communities [HMSC (84, 85)] to analyze the effect of the different intrinsic factors (sex, reproductive age, behavior, and MORV infection history by using the presence of MORV-specific antibodies) on infection probability and load of the different helminths (86). Hierarchical Modeling of Species Communities is a joint species distribution model which includes a hierarchical layer which investigates how species respond to environmental covariates depending on different species traits and phylogenetic relationships (87, 88).

The data we used as response variables [the matrix *n* × *n*_*s*_ Y of HMSC; see (84, 85)] consisted of the parasite load of the different gastrointestinal helminths which were found in the 214 individuals (see section Results). We excluded two rare parasitic genera which occurred in less than three individuals (see section Results). We had to apply a hurdle model to account for the zero-inflation in our data. This means that we had to create two different models: (1) a presence-absence model and (2) an abundance model conditional on the presence of the parasite [abundance COP model (85)]. The response variables were transformed to a binomial variable in the presence-absence model where the parasite was either present (1) or absent (0) within a certain individual. This model allowed us to investigate which intrinsic factor affected the probability that a certain individual becomes infected with a certain parasite. In the second model (abundance COP model) we log transformed the count data of the different gastrointestinal parasites and treated the absence of a certain parasite, within an individual, as missing data and are thus ignored in this model. This model allowed us to study the effect of the different factors on the parasite load after the individual became infected and is therefore independent of the infection probability. This model allowed us to look at potential factors that affected the reinfection rate of certain parasites, depending on the transmission cycle of the parasite.

We considered the individuals' idsentity as the sample unit which was then subsequently nested as a random effect within the site in which the individual was trapped (which was added as another random effect) in both models. As fixed effects [the *n* × *n*_*c*_ matrix X of HMSC; see (84, 85); where *n*_*c*_ is the number of individual-specific regression parameters to be estimated], we included the individuals' sex (male or female), reproductive age (adult or juvenile), their exploration, and stress-sensitivity behavior, expressed in the hole-board test and their infection history with the MORV using the presence of MORV specific antibodies [MORVab; (68)]. The two behavioral measurements were the residuals derived from the LMMs as described above. Additionally, we included the transmission mode of the parasite (direct or indirect) as a species trait in both models.

Both HMSC models were fitted with the R-package Hmsc [version 3.0-9 (89)] using the default prior distributions (85). We sampled the posterior distribution with five Markov Chain Monte Carlo (MCMC) chains, each of which was run with 3,000,000 iterations of which the first 1,000,000 were removed as burn-in. The chains were thinned by 1,000 to yield 2,000 posterior samples per chain which resulted in 10,000 posterior samples in total. We examined MCMC convergence using the potential scale reduction factors of the model parameters (85). The explanatory and predictive power of the presence-absence model was examined using the species-specific AUC (90) and Tjur's *R*^2^ (91) values. The explanatory and predictive powers of the abundance COP models were measured by *R*^2^. Explanatory power was computed by making model predictions based on models which were fitted to all data. Predictive power was computed by performing a five-fold cross-validation, in which the sampling units were assigned randomly to five-folds, and predictions for each fold were based on model fitted to data on the remaining four-folds.

## Results

### Behavioral Analysis

The PCA reduced the number of behavioral variables to two, explaining 70.4% of the total variance (Table 1). The first component was positively correlated with activity and the two measurements of exploration: head dipping and hole sniffing (Table 1). The second component was positively correlated with jumping and negatively with self-grooming (Table 1). Both axes correspond to the previous results of Vanden Broecke et al. (59) who used the same behavioral setup on *M. natalensis*, and referred to the first component as exploration and the second component was referred to as stress-sensitivity. Since we found the same behavioral axes, we decided to adopt the same names for the two components which we will further refer to. We do note that both traits can be seen as personality traits, since Vanden Broecke et al. (59) found that there were consistent among-individuals differences through time for both exploration (repeatability = 0.22) and stress-sensitivity (repeatability = 0.44).

**Table 1:** Correlation of each behavior observed during the hole-board test with the axes resulting from the principal component analysis.

**Component**	**PC1 (exploration)**	**PC2 (stress-sensitivity)**
Activity	**0.528**	−0.119
Head dip	**0.502**	−0.292
Sniffing	**0.569**	−0.234
Grooming	−0.242	–**0.694**
Jumping	0.295	**0.604**
Total variance (%)	0.488	0.217
Eigenvalue	2.438	1.083

*Bold type indicates the behaviors that had a major contribution to a component*.

The LMM with exploration as response variable revealed that the interaction between sex and age was not significant [coefficient ± SE = 0.042 ± 0.533, *t*_(199)_ = 0.078, *P* = 0.938]. Males were not significantly more explorative than females [coefficient ± SE = 0.387 ± 0.250, *t*_(207)_ = 1.547, *P* = 0.123] and juveniles did not differ from adults regarding their explorative behavior expressed in the hole-board test [0.263 ± 0.339, *t*_(203)_ = 0.775, *P* = 0.439]. The interaction between sex and age was significant in the LMM with stress-sensitivity as response variable [0.675 ± 0.335, *t*_(201)_ = 2.015, *P* = 0.045] where only juvenile males were significantly less stress-sensitive compared to adult males [−0.945 ± 0.261, *t*_(203)_ = −3.614, *P* = 0.002] and adult females [−1.017 ± 0.246, *t*_(204)_ = −4.135, *P* < 0.001], while there we no differences between the other classes (*P* > 0.05).

### Parasitology

Of the 214 individuals that were trapped during this study, 166 (78%) were infected with at least one helminth. We found eight different helminth genera infecting *M. natalensis*: five nematodes and three cestodes. The different nematode genera were: *Protospirura muricola* [*N*_infected individuals(IF)_ = 75, prevalence = 35.05%, mean parasite load = 14.69; range = 1–131], *Syphacia* sp. (*N*_IF_ = 45, prevalence = 21.03%, mean parasite load = 33.24; range = 1–283), *Trichuris mastomysi* (*N*_IF_ = 30, prevalence = 14.02%, mean parasite load = 5.30; range = 1–30), *Gongylonema* sp. (*N*_IF_ = 2, prevalence = 0.93%, mean parasite load = 4; range = 3–5), and *Pterygodermatites* sp. (*N*_IF_ = 1, prevalence = 0.5%, parasite load = 2). *Protospirura muricola* is a stomach nematode which primarily infects murid rodents (92). The worms can reach more than 5 cm in length and often accumulate in the host resulting in a high worm burden (93, 94). *Protospirura muricola* needs an intermediate, arthropod host to complete its life-cycle (92). Both *Syphacia* sp. and *Trichuris mastomysi* can be found in the caecum of the host and do not need a secondary host (95, 96). *Gongylonema* sp. resides in the esophageal mucosa of their final host, but they need an arthropod as intermediate host in order to complete their life-cycle. *Pterygodermatites* sp. has a similar life-cycle, but they infect the small intestines of their final host (95, 96).

The cestode genera were: *Hymenolepis* sp. *(N*_IF_ = 97, prevalence = 45.33%, mean parasite load = 5.56; range = 1–98), *Raillietina* sp., and *Inermicapsifer* sp. However, the last two were not always distinguishable from each other and we therefore decided to pool their counts at the family level: *Davaineidae* (*N*_IF_ = 60, prevalence = 28.04%, mean parasite load = 4; range = 1–15). All three genera infect the small intestines of their final host and they all need an arthropod as an intermediate host in order to complete their life-cycle (95, 97).

Additionally, we found one other nematode family: *Trichostrongylidae* (*N*_IF_ = 30, prevalence = 14.02%, mean parasite load = 18.90; range = 1–124) for which we could not separate the different genera from each other. Member of this family occupy the small intestine of their host. The different larval stages that follow after the eggs are being passed out of their host are free-living and employ a questing behavior which increases their chance of being eaten by another host (96).

### Hierarchical Modeling of Species Communities

The MCMC convergence of the two HMSC models was satisfactory: the potential scale reduction factors for the β-parameters [which measure the responses of the helminth species to the different intrinsic covariates (84)] was on average 1.001 (max = 1.004) for the presence-absence model and 1.000 (max = 1.001) for the abundance COP model. The presence-absence model fitted the data adequately, with a mean AUC of 0.880 (0.752–0.992) for the explanatory power and 0.636 (0.525–0.798) for the predictive power (Supplementary Figure 2A). The mean Tjur *R*^2^ for the explanatory power was on average 0.223 (0.067–0.371) and 0.079 (0.004–0.229) for the predictive power (Supplementary Figure 2B). This means that the model explained and predicted the data better than by random (85). The explanatory power of the abundance COP model was high, with a mean *R*^2^ of 0.526 (0.363–0.766) while the predictive power was quite low with a mean *R*^2^ of 0.004 (−0.025–0.034; Supplementary Figure 3) suggesting that the results from this model are less reliable compared to the presence-absence model.

**Figure 1:**
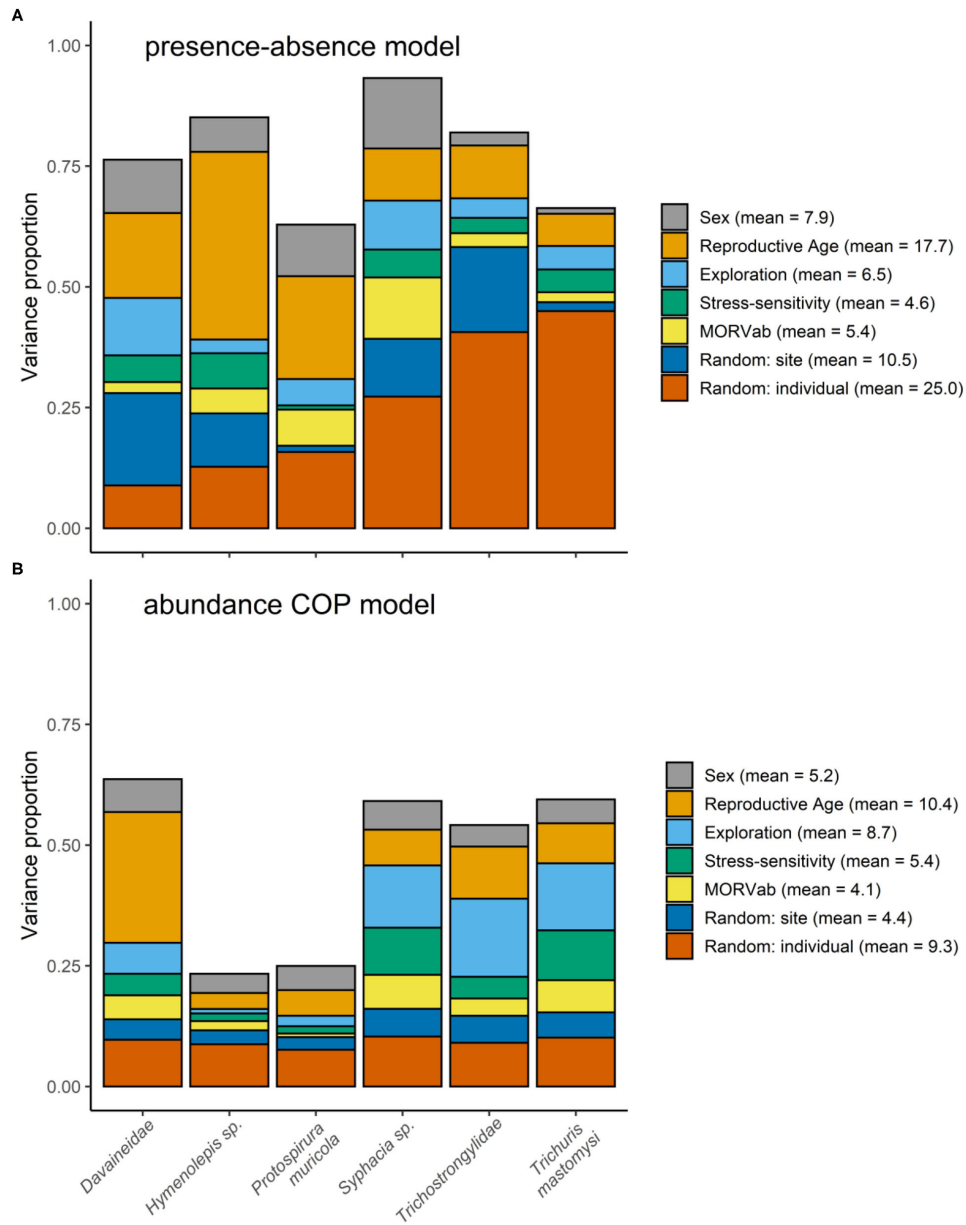
Variance partitioning among the different fixed and random effects from the **(A)** presence-absence model and **(B)** abundance COP model. The height of the bars in both panels correspond to the explanatory power achieved by for the different helminth species, measured by the Tjur *R*^2^ for the presence-absence model and *R*^2^ for the abundance COP model. The legend give the mean variance proportions for each fixed and random effect within the model, averaged over the different helminths.

**Figure 2:**
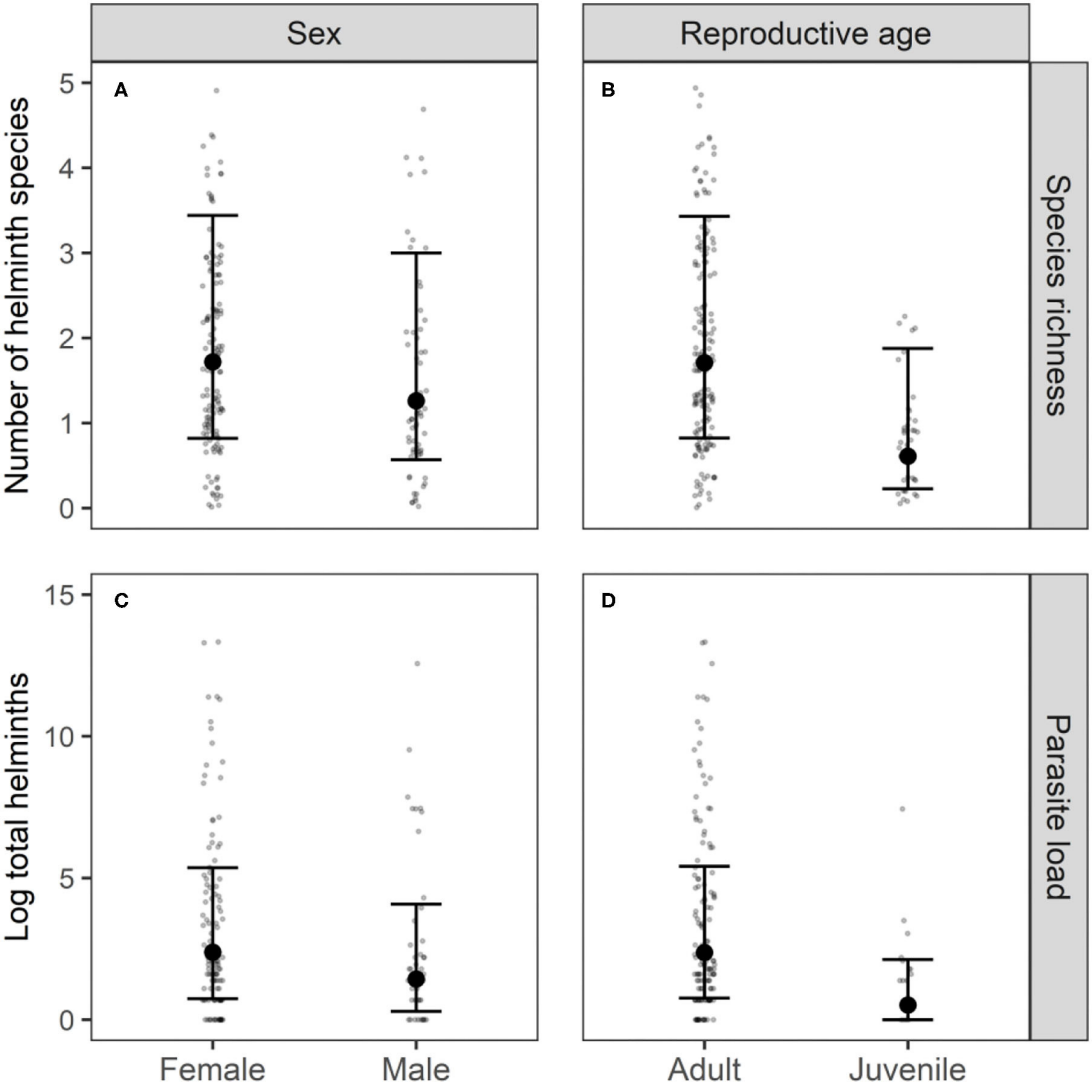
Differences in species richness and log-transformed parasite load between sexes **(A,C)** and reproductive age groups **(B,D)**. Black dots and error bars are, respectively, the predicted means and 95% credible intervals derived from the presence-absence model **(A,B)** and abundance COP model **(C,D)**. Gray dots are the observations per individual.

Variance partitioning showed that the hosts' age explained a substantial amount of the variation in the presence-absence model (17.7%; Figure 1A) and in the abundance COP model (10.4%; Figure 1B). Indeed, the presence-absence model revealed that the helminth species richness was higher in adults [mean = 1.71; 95% CI: (0.83–3.43)] compared to juveniles [mean = 0.61; 95% CI: (0.23–1.88); Figure 2B], since juveniles were less likely to become infected with *T. mastomysi, Trichostrongylidae, P. muricola, Davaineidae*, and *Hymenolepis* sp. (Table 2; Supplementary Figure 4). Additionally, the abundance COP model revealed that the parasite load was higher in adults [mean = 2.37; 95% CI: (0.77–5.42)] compared to juveniles [mean = 0.52; 95% CI: (0.00–2.13); Figure 2D], which was mainly driven by *Davaineidae* in which the parasite load was significantly lower in juveniles compared to adults (Table 3; Supplementary Figure 5).

**Table 2:** Posterior mean responses (with their 95% credibility intervals) of the different helminth species to the fixed effects derived from the presence-absence model.

	**Presence-absence model**					
	***Hymenolepis sp*.**	***Davaineidae***	***Protospirura muricola***	***Trichostrongylidae***	***Trichuris mastomysi***	***Syphacia sp*.**
**Fixed effect**	**est (95% CrI)**	**est (95% CrI)**	**est (95% CrI)**	**est (95% CrI)**	**est (95% CrI)**	**est (95% CrI)**
Intercept	0.05 (−0.24; 0.33)	–**0.36 (**–**0.74; 0.05)**	−0.16 (−0.47; 0.14)	–**1.25 (**–**1.81;** –**0.82)**	–**1.54 (**–**2.35;** –**0.98)**	–**0.99 (**–**1.30;** –**0.70)**
Sex (male)	−0.27 (−0.70; 0.11)	–**0.63 (**–**1.08;** –**0.20)**	–**0.85 (**–**1.40;** –**0.39)**	−0.01 (−0.52; 0.46)	−0.04 (−0.65; 0.53)	0.31 (−0.08; 0.70)
Age (juvenile)	–**0.88 (**–**1.54;** –**0.42)**	–**0.94 (**–**1.54;** –**0.40)**	–**1.44 (**–**2.18;** –**0.83)**	–**0.55 (**–**1.19; 0.04)**	–**0.76 (**–**1.59;** –**0.08)**	−0.27 (−0.76; 0.19)
Exploration	0.02 (−0.17; 0.21)	–**0.31 (**–**0.55;** –**0.10)**	–**0.29 (**–**0.53;** –**0.07)**	−0.09 (−0.35; 0.15)	–**0.26 (**–**0.61; 0.04)**	−0.10 (−0.31; 0.09)
Stress-sensitivity	0.13 (−0.06; 0.34)	–**0.19 (**–**0.42; 0.01)**	−0.05 (−0.26; 0.17)	−0.02 (−0.27; 0.23)	−0.25 (−0.60; 0.06)	−0.01 (−0.21; 0.18)
MORVab (P)	0.27 (−0.19; 0.78)	0.29 (−0.20; 0.77)	**0.91 (0.39; 1.49)**	0.12 (−0.47; 0.72)	0.36 (−0.29; 1.06)	0.36 (−0.13; 0.83)

*Estimates with 95% posterior support are marked in bold*.

**Table 3:** Posterior mean responses (with their 95% credibility intervals) of the different helminth species to the fixed effects derived from the abundance COP model.

	**Abundance COP model**					

	***Hymenolepis sp***.	***Davaineidae***	***Protospirura muricola***	***Trichostrongylidae***	***Trichuris mastomysi***	***Syphacia sp***.
**Fixed effect**	**est (95% CrI)**	**est (95% CrI)**	**est (95% CrI)**	**est (95% CrI)**	**est (95% CrI)**	**est (95% CrI)**
Intercept	0.07 (−0.21; 0.34)	0.04 (−0.26; 0.35)	0.04 (−0.26; 0.33)	−0.05 (−0.47; 0.38)	−0.08 (−0.51; 0.35)	0.03 (−0.36; 0.42)
Sex (male)	–**0.39 (**–**0.79; 0.01)**	−0.24 (−0.75; 0.30)	–**0.46 (**–**0.98; 0.05)**	0.11 (−0.52; 0.74)	−0.01 (−0.65; 0.64)	−0.19 (−0.71; 0.32)
Age (juvenile)	−0.37 (−0.94; 0.21)	–**0.78 (**–**1.51;** –**0.05)**	−0.53 (−1.40; 0.37)	−0.51 (−1.36; 0.33)	−0.26 (−1.19; 0.71)	−0.28 (−0.92; 0.36)
Exploration	0.00 (−0.19; 0.19)	−0.08 (−0.33; 0.17)	−0.12 (−0.34; 0.11)	–**0.29 (**–**0.60; 0.01)**	−0.22 (−0.64; 0.20)	−0.19 (−0.48; 0.09)
Stress-sensitivity	−0.08 (−0.27; 0.11)	0.03 (−0.24; 0.28)	−0.06 (−0.28; 0.16)	0.03 (−0.28; 0.34)	0.16 (−0.27; 0.58)	0.16 (−0.10; 0.43)
MORVab (P)	0.30 (−0.15; 0.74)	0.24 (−0.28; 0.76)	0.03 (−0.43; 0.48)	0.12 (−0.60; 0.82)	0.34 (−0.34; 1.03)	0.35 (−0.23; 0.92)

*Estimates with 95% posterior support are marked in bold*.

The host's sex was responsible for 7.9% of the variation in the presence-absence model (Figure 1A) and 5.2% in the abundance COP model (Figure 1B). Species richness was slightly lower in males [mean = 1.26; 95% CI: (0.57–3.00)] compared to females [mean = 1.72; 95% CI: (0.82–3.44); Figure 2A] since males were less likely to become infected with helminths with an indirect life cycle compared to females (posterior mean = −0.576; posterior support = 0.921). This effect was mainly driven by two helminth species, since males had a significantly lower infection probability than females for *Davaineidae* and *P. muricola* (Table 2; Supplementary Figure 6). The abundance COP model showed that the parasite load of *Hymenolepis* sp. and *P. muricola* was significantly lower in males than females as well (Table 3; Supplementary Figure 7), which resulted in an overall lower parasite load in males [mean = 1.44; 95% CI: (0.30–4.08)] compared to females [mean = 2.39; 95% CI: (0.74–5.36); Figure 2C].

**Figure 3:**
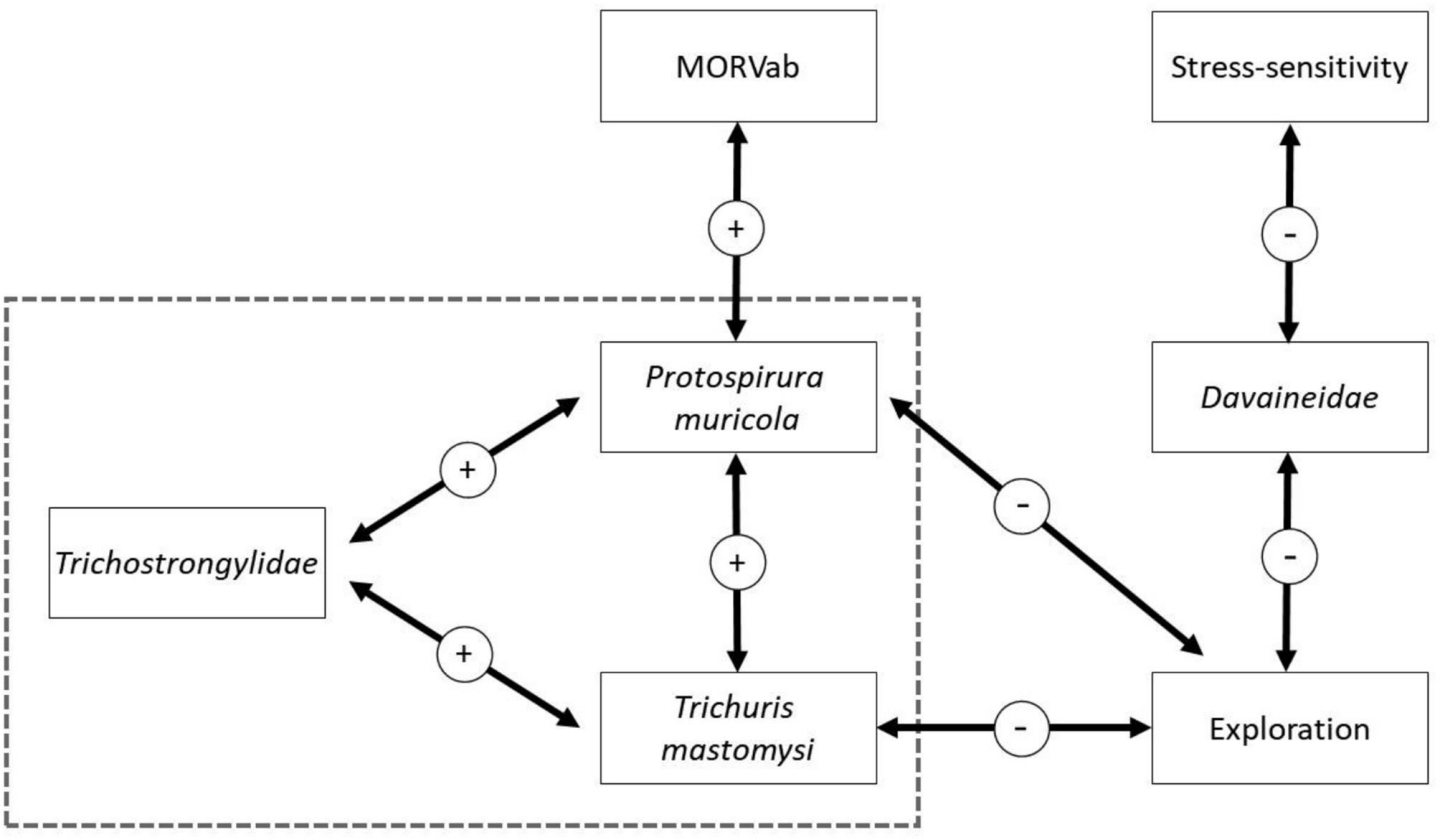
Graphical summary of the results from the presence-absence HMSC model. Arrows indicate strong correlation, with 95% posterior probability support, between the two variables. Arrows within the dashed square are derived from the co-occurrence results (the omega parameters) while the arrows outside are derived from the beta estimates. The sign of the estimate is given in the circle.

**Figure 4:**
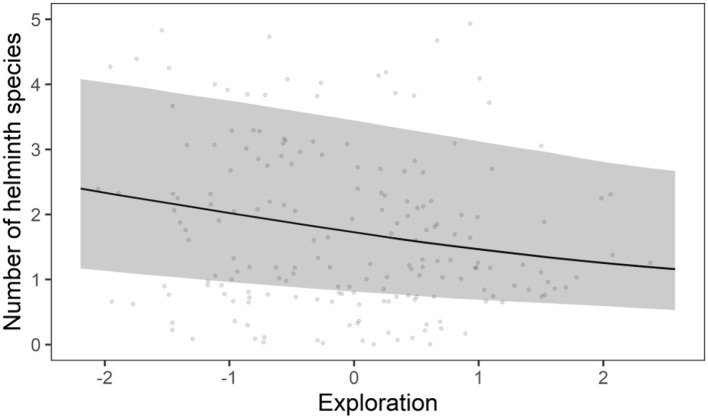
Correlation between exploration and helminth species richness derived from the presence-absence model. The line represent the predicted correlation, while the shaded band represents the 95% credible interval. The points are the observed values per individual.

The HMSC variance partitioning also revealed that the hosts' behavior was responsible for 11.1% of the variation in the presence-absence model (exploration = 6.5%; stress-sensitivity = 4.6%; Figure 1A) and 14.1% in the abundance COP model (exploration = 8.7%, stress-sensitivity = 5.4%; Figure 1B). The presence-absence model revealed a negative correlation between exploration behavior and infection probability for *Davaineidae, P. muricola*, and *T. mastomysi* (Table 2; Supplementary Figure 8 and simplified in Figure 3), which suggests that less explorative individuals were more likely to become infected with these worms resulting in a negative correlation between helminth richness and exploration behavior (estimate = −0.26; Figure 4). However, we did not find the same relationship with parasite load (Table 3). Additionally, while exploration behavior did not affect infection risk of *Trichostrongylidae* (Table 2), it did affect their parasite load. Indeed, the abundance COP model revealed a negative correlation between exploration and parasite load of *Trichostrongylidae* (Table 3; Supplementary Figure 9), suggesting that less explorative individuals had a higher parasite load of *Trichostrongylidae* compared to highly explorative individuals. Stress-sensitivity had a negative effect on infection probability with *Davaineidae* (Table 2; Supplementary Figure 10 and simplified in Figure 3), which suggests that more stress-sensitive individuals, who jumped more frequently during the hole-board test were less likely to become infected with *Davaineidae*. However, this effect was not strong and was influenced by some extreme values, since this effect disappeared when we left them out of the analysis. We decided, however, to keep these individuals in the analysis in order to keep our sample size as large as possible and because these extreme values were only present in the stress-sensitivity variable.

Lastly, we found that individuals with antibodies against the MORV were significantly more likely to be infected with *P. muricola* (Table 2; Supplementary Figure 11 and simplified in Figure 3). Nonetheless, the host's identity, which we included as a random effect in our model explained the largest part of the variation in both the presence-absence model (25.5%; Figure 1A) as in the abundance COP model (9.4%; Figure 1B), which suggests that that a large proportion of the variance can be ascribed to unaccounted variation among the individuals.

Finally, the presence-absence model revealed that *P. muricola, T. mastomysi*, and *Trichostrongylidae* showed a positive co-occurrence pattern, with 95% posterior probability support, within the host after controlling for host-associated (sex, age, behavior, and MORVab) and spatial confounding factors (Supplementary Table 3 and simplified in Figure 3). However, this effect was only present in the presence-absence model and not in the abundance COP model (Supplementary Table 5), which suggests that even though these three parasites occur more frequently together within the host than would be expected, this was not true for parasite loads. We found no co-occurrence among the different helminths among the different sites in which they were trapped (Supplementary Tables 2, 4), which suggests that there were no areas where certain parasites co-occurred more frequently together than would be expected by random. Nonetheless, the site in which the individual, was trapped was responsible for 10.5% of the variation on the presence-absence model (Figure 1A) and 4.4% on the abundance COP model (Figure 1B). This may suggest that unmeasured variation among the different sites affects the presence of certain helminths. Alternatively, it is possible that this is a results of the variation in the trapping effort among the different sites.

## Discussion

In this study, we investigated the effect of different intrinsic factors (i.e., the host's sex, age, behavior, and viral infection history) on the infection risk and parasite load of the whole gastrointestinal helminth community in *M. natalensis*. In general, we found that adults were more likely to become infected with almost all parasite species resulting in a higher species richness in adults compared to juveniles. Additionally, we found that females had a higher infection risk and parasite load compared to males. Lastly, we found a correlation between species richness and the host's behavior, but the direction was opposite to our initial hypothesis. We found that less explorative individuals had a higher infection probability of three parasitic species, which resulted in a negative correlation between exploration behavior and helminth richness.

### Parasite Prevalence

We found eight different helminth genera and one family during this study, but the prevalence varied strongly among them. The two most dominant gastrointestinal helminths species in our study were *Hymenolepis* sp. and *P. muricola*. These results correspond with other studies performed on the African continent, since both species are widespread in this part of the world and are able to infect a variety of murid rodents with a high prevalence (57, 92, 93, 98). The third most abundant group in our study was the *Davaineidae* family. It is difficult to compare this with other studies since we grouped two genera together (i.e., *Raillietina* sp. and *Inermicapsifer* sp.) into one family and because the prevalence can vary seasonally. Fichet-Calvet et al. (99), for instance, showed that the prevalence of *Raillietina trapezoids* infection in the fat sand rat (*Psammomys obesus*) varied seasonally from 2% in spring to 100% in late autumn which may explain the high variation in prevalence found in other studies as well [see (52) and (67)].

*Syphacia* sp., *Trichuris mastomysi*, and the *Trichostrongylidae* family had an intermediate prevalence. These three nematodes have a direct life cycle, meaning that they do not need a secondary host in their development. These results are in contrast with other work in Senegal and South Africa, where helminths with a direct life cycle were the most dominant species (52, 100). This may be explained by differences in environmental conditions, which are known to play a key role in the survival of helminths during their free-living stages (5–8). *Gongylonema* sp. and *Pterygodermatites* sp. had the lowest prevalences of <1% which corresponds with previous work in Senegal and South Africa (52, 57, 98, 101).

### Age and Sex Effects

Our results revealed a strong age effect on the parasitic infection probability, where adults were more likely to be infected with all helminths, except for *Syphacia* sp., which resulted in a higher parasitic diversity in adults compared to juveniles. This is a common pattern and can be ascribed to the higher energetic needs in adults and a prolonged exposure time to infectious environments and/or secondary hosts (52, 67, 102). These secondary hosts are mainly invertebrates (95, 96) and the consumption of invertebrates is not that unusual in *M. natalensis*, due to their opportunistic and generalist diet (64, 75, 103–105). Adults had indeed more time to come into contact with these secondary hosts because our fieldwork was performed during the breeding seasons of *M. natalensis* (71, 72). This means that all the juveniles that were trapped during this study were born within the same year and had therefore fewer opportunities to come into contact with infected materials and/or infected secondary hosts compared to adults, resulting in lower parasite diversity. This increased infection probability of helminth infections in adults, however, did not translate into higher parasite loads, since we found no significant differences in parasite load between adults and juveniles after they became infected. This could be due to an immunological response or due to intraspecific [such as the crowding effect (94, 99)] and/or interspecific competition among parasites (see below). The only exception was the *Davaineidae* family, where adults had a higher infection probability and load compared to juveniles, but the exact reason for this is not clear.

Besides the age effect, we found that females were infected with a slightly wider variety of helminth species compared to males, which was mainly driven by *P. muricola* and the *Davaineidae* family. This pattern contradicts the common male-bias of helminth infections in mammals, which is believed to be caused by the immunosuppressive effect of testosterone (65, 66). However, this is not a general rule. Rossin et al. (102), for instance, found that female Talas tuco-tuco (*Ctenomys talarum*) had a higher prevalence of the *Strongyloides myopotami* nematode compared to males, potentially because they spent more time in their burrows. This, however, might not be the case in *M. natalensis*, even though females spent more time near and in their burrows than males during the breeding season (63). We hypothesize that this sex effect could be attributed to dietary differences between the two sexes. Indeed, our models showed that females were more likely to become infected with helminths with an indirect lifecycle and females had a significant higher parasite load for both *Hymenolepis* sp. and *P. muricola*. This may suggest that females consume more invertebrates compared to males since the secondary hosts of these parasites are invertebrates (95, 96). This may arise due to the higher energetic demand for proteins in females since most females are either pregnant or need to care for their litters during the breeding seasons.

### Co-Infection Patterns and Behavior

Our models revealed a positive association between *P. muricola, T. mastomysi*, and *Trichostrongylidae* infections within an individual (Figure 3). This suggests that these three helminths co-occur more frequently together than expected by random. One potential explanation for this result is that infection with one of these nematodes reduces the host's immunity, which makes them more vulnerable to subsequent helminth infections (11). Nonetheless, these co-infection pattern did not lead to higher loads of these three nematodes, either because such burdens are fatal (106, 107) and are therefore not present in our dataset or because there is some sort of density-dependent competition within (94) and/or among these three species. Indeed, while all three nematodes occupy different parts inside the gastrointestinal tract [*P. muricola* in the stomach, *T. mastomysi* in the cecum, and *Trichostrongylidae* in the small intestines (95, 96)] they are all dependent on the same resources which may prevent them from reaching high abundancies.

An alternative, non-mutually exclusive, explanation for this co-infection pattern is that the infection risk of these parasites are affected by the same risk behavior of the host, since we found that less explorative individuals were more likely to become infected with *P. muricola, T. mastomysi*, and *Davaineidae* (Figure 3) and had a significantly higher parasite load of *Trichostrongylidae*. This, however, is the opposite of what we hypothesized, since we predicted that highly explorative individuals would have a higher parasitic species richness and load compared to less explorative individuals. A potential explanation is that less explorative individuals have an increased exposure rate to these helminths due to a larger spatial activity pattern. Most of the studies that found a positive correlation between exploration or boldness and endoparasite infection risk used trappability (the number of times an individual was trapped) as a personality trait instead of the behaviors expressed in an open field arena (27, 28, 30). Trappability is associated with space use in several rodents species (25, 30, 58) and may therefore suggest that individuals who are more active have a higher risk to become infected due to a larger exposure rate to different infected environments. Nonetheless, the link between exploration, measured in an open field arena and trappability is not always clear or present (108–110). Vanden Broecke et al. (58, 79), found no correlation between exploration and trappability in *M. natalensis* which may suggest that this explanation is not very likely. Alternatively, while exploration might not affect exposure rate, it is possible that less explorative individuals are more susceptible to become infected after they came into contact with infected materials [feces or secondary hosts (13–15)]. Indeed, Kortet et al. (111) argued that immunologically competent individuals should be bolder and more explorative than less competent individuals, which increases their resistance against parasitic infections. This has been confirmed in house finches (*Haemorhous mexicanus*), where highly explorative individuals had a better innate immune system compared to less explorative individuals (112). Similar results have been found in the collared flycatcher (*Ficedula albicollis*) where individuals with more MHC alleles and an efficient immune system take more risks (113).

However, we should note that our models did not allow us to determine the direction of causality. It is therefore possible that the helminth infection, by itself, is responsible for the reduction of the host's exploration behavior, which is not unlikely (24, 111, 114). Indeed, a higher parasite burden inside the host might lead to a lower energy uptake of the host. This may then result in a lower mobility during the hole-board test, since the host has to share its resources with its intestinal parasites, especially when these parasites occupy different parts inside the gastrointestinal tract (95, 96). Such an effect has been found in lab mice, for instance, where individuals who were infected with *Trichinella spiralis* or *Hymenolepis fraternal* became less active inside an open-field test (115). In this study, *P. muricola* infection in particular may have a strong impact on the resource availability of our study species. *P. muricola* infection in spiny mice (*Acomys cahirinus dimidiatus*), for instance, has been found to lead to heavy worm burdens in their stomach (>2% of the host body weight) with detrimental fitness effects (93, 94). But a large worm burden does not necessarily mean a higher parasite load since the length and weight of the parasites plays an additional role and in turn may covary with the number of parasites that are present inside the host. Indeed, Lowrie et al. (94) found that the growth of *P. muricola* was density-dependent where the length and weight of the worms decreased with the number of parasites, which could potentially explain that we found no correlation between exploration and *P. muricola* load.

If *P. muricola* infection does indeed reduce the availability of resources to the host, it could make the host more vulnerable to additional helminth infections (leading to co-infection patterns) and viral infections. Indeed, we have found that individuals with antibodies against the MORV were significantly more likely to be infected with *P. muricola* (Figure 3) which could affect the individuals fitness. Mariën et al. (70) found that MORVab positive individuals had a lower survival probability compared to MORVab negative individuals. We could, however, not disentangle the sequence of infection in our study but Mariën et al. (70) suggested that the lower survival probability of MORVab positive individuals could be due to adverse effects of MORV infection on the individual's health. Indeed, even though infected individuals recover quickly from MORV infection (69), their body condition has been found to decrease shortly after infection (116) which could make them more vulnerable for helminth infections. But this period is quite short and can therefore not fully explain the link between *P. muricola* and MORVab presence. A more plausible explanation that they propose is that MORV infection could be seen as a secondary infection due to poorer body conditions, potentially because of the aggregation of gastrointestinal parasites inside the host, as has been found in field voles [*Microtus agrestis* (117)]. This explanation may also suggest that the negative correlation between exploration and MORVab presence, found in Vanden Broecke et al. (59), is potentially indirect, where less explorative individuals are more likely to become infected with *P. muricola*, which reduces their immune system making them more vulnerable to MORV infection. Nonetheless, experimental studies are needed to confirm this link between *P. muricola* and MORV infection and should take other factors, such as population density, sequence of infection and immunological responses to helminth and MORV infection into account (58, 68, 118).

## Data Availability Statement

The datasets presented in this study can be found in online repositories. The names of the repository/repositories and accession number(s) can be found at: doi 10.17605/OSF.IO/QJXNU.

## Ethics Statement

The animal study was reviewed and approved by the University of Antwerp Ethical Committee for Animal Experimentation (2016-63), adhered to the EEC Council Directive 2010/63/EU, and followed the Animal Ethics guidelines of the Research Policy of the Sokoine University of Agriculture.

## Author Contributions

BVB, LB, AR, LM, EM, and HL designed the study. LB performed the fieldwork with support from BVB and LM. Parasitological examination was done by LB and AR. The blood samples were analyzed by BVB. BVB and VS performed the data analysis with support from LB, EM, and HL. The first draft was written by BVB and LB. All authors contributed substantially to revisions.

## Conflict of Interest

The authors declare that the research was conducted in the absence of any commercial or financial relationships that could be construed as a potential conflict of interest.

## Publisher's Note

All claims expressed in this article are solely those of the authors and do not necessarily represent those of their affiliated organizations, or those of the publisher, the editors and the reviewers. Any product that may be evaluated in this article, or claim that may be made by its manufacturer, is not guaranteed or endorsed by the publisher.
