# Supplementary material for: Linking Behavior, Co-infection Patterns, and Viral Infection Risk With the Whole Gastrointestinal Helminth Community Structure in Mastomys natalensis
Source: Front Vet Sci. 2021 Aug 17;8:669058. doi: 10.3389/fvets.2021.669058 (PMC8415832; doi:10.3389/fvets.2021.669058)

Supplementary information

Figure 1: the eleven different sample sites (yellow circles) on the campus of the Sokoine University of Agriculture in Morogoro, Tanzania.

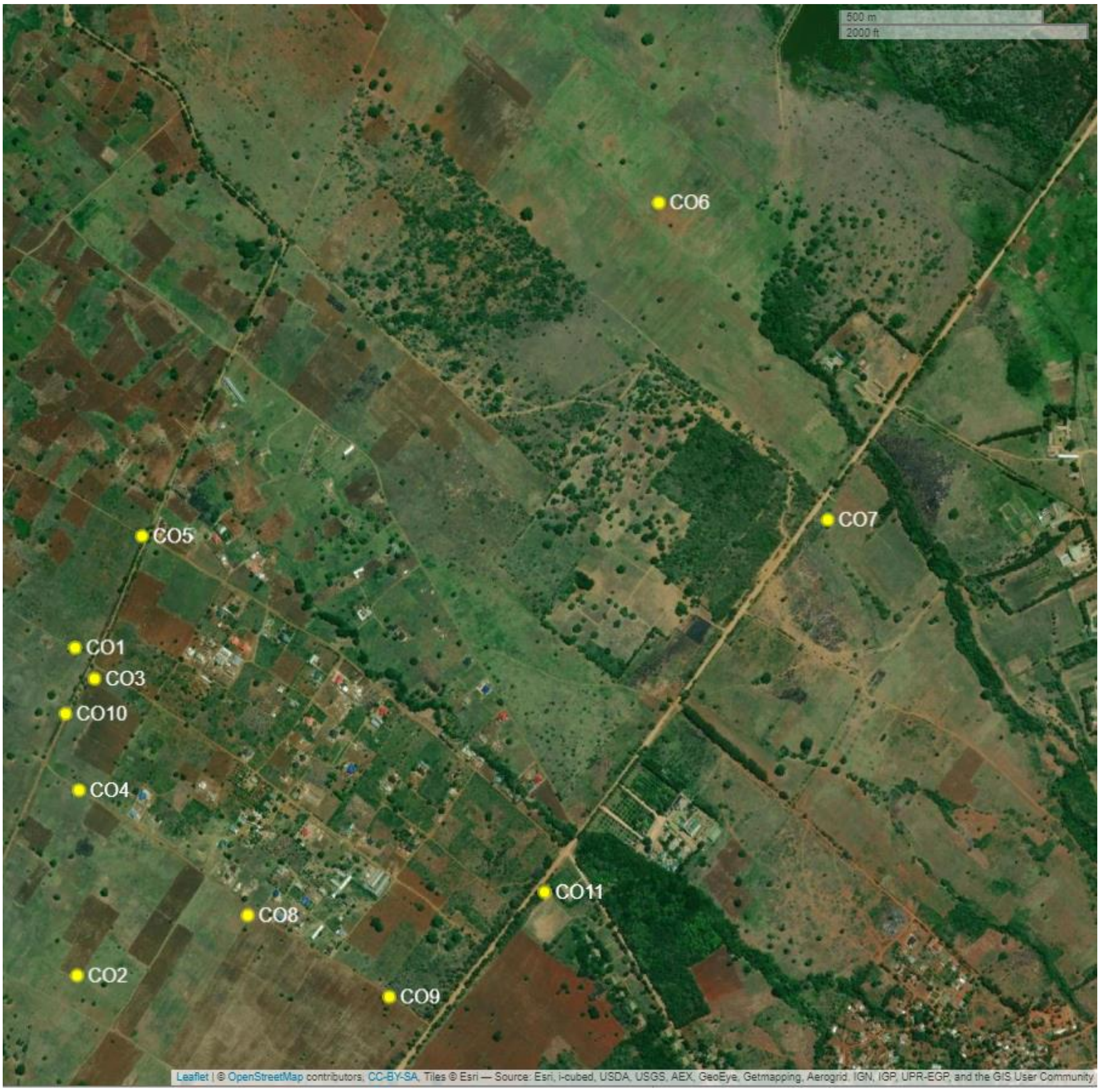

Table 1: The number of traps which were set in each site per night.

Site	Date	Traps
CO1	15/08/2019	100
	21/08/2019	100
CO2	15/08/2019	100
CO3	25/07/2019	100
	26/07/2019	200
	01/08/2019	150
	02/08/2019	150
	04/08/2019	150
CO4	06/08/2019	150
	07/08/2019	140
	08/08/2019	150
	09/08/2019	150
CO5	13/08/2019	150
	14/08/2019	100
	22/08/2019	100
CO6	28/08/2019	140
	29/08/2019	100
CO7	03/09/2019	150
CO8	04/09/2019	140
	05/09/2019	80
	10/09/2019	100
	12/09/2019	100
	18/09/2019	150
CO9	17/09/2019	150
CO10	19/09/2019	150
	25/09/2019	150
	26/09/2019	150
CO11	03/09/2019	100

Table 2: Co-occurrence (posterior mean and support) of the different helminths within the trapping fields based on the presence-absence model after controlling for host-associated factors (sex, age, behavior and MORVab).

Co-occurence within trapping site: presence-absence model					
	Davaineidae	*Protospirura muricola*	*Trichostrongylidae*	*Trichuris mastomysi*	*Syphacia* sp.
	mean (sup.)	mean (sup.)	mean (sup.)	mean (sup.)	mean (sup.)
Hymenolepis sp.	-0.38 (0.30)	-0.15 (0.42)	-0.18 (0.40)	-0.18 (0.40)	-0.24 (0.37)
Davaineidae		0.53 (0.78)	-0.39 (0.29)	0.51 (0.77)	0.53 (0.78)
Protospirura muricola			-0.32 (0.33)	0.34 (0.68)	0.31 (0.67)
Trichostrongylidae				-0.27 (0.36)	-0.25 (0.36)
Trichuris mastomysi					0.37 (0.70)

Table 3: Co-occurrence (posterior mean and support) of the different helminths within an individual based on the presence-absence model after controlling for host-associated factors (sex, age, behavior and MORVab). Co-occurrences with a 95% posterior probability support are marked in bold.

Co-occurence within the individual: presence-absence model					
	Davaineidae	*Protospirura muricola*	*Trichostrongylidae*	*Trichuris mastomysi*	*Syphacia* sp.
	mean (sup.)	mean (sup.)	mean (sup.)	mean (sup.)	mean (sup.)
Hymenolepis sp.	0.34 (0.69)	0.56 (0.79)	0.52 (0.77)	0.56 (0.79)	0.48 (0.76)
Davaineidae		0.76 (0.89)	0.79 (0.91)	0.76 (0.89)	0.65 (0.84)
Protospirura muricola			**0.92 (0.97)**	**0.96 (0.98)**	0.81 (0.92)
Trichostrongylidae				**0.93 (0.97)**	0.79 (0.91)
Trichuris mastomysi					0.81 (0.92)

Table 4: Co-occurrence (posterior mean and support) of the different helminths within the trapping fields based on the abundance COP model after controlling for host-associated factors (sex, age, behavior and MORVab).

Co-occurence within trapping site: abundance COP model					
	Davaineidae	*Protospirura muricola*	*Trichostrongylidae*	*Trichuris mastomysi*	*Syphacia* sp.
	mean (sup.)	mean (sup.)	mean (sup.)	mean (sup.)	mean (sup.)
Hymenolepis sp.	0.05 (0.53)	0.49 (0.76)	0.12 (0.56)	-0.17 (0.41)	-0.23 (0.38)
Davaineidae		0.05 (0.53)	0.13 (0.57)	0.05 (0.53)	-0.05 (0.47)
Protospirura muricola			0.13 (0.57)	-0.14 (0.42)	-0.24 (0.38)
Trichostrongylidae				0.08 (0.54)	-0.10 (0.45)
Trichuris mastomysi					0.05 (0.53)

Table 5: Co-occurrence (posterior mean and support) of the different helminths within an individual based on the abundance COP model after controlling for host-associated factors (sex, age, behavior and MORVab).

Co-occurence within individuals: abundance COP model					
	Davaineidae	*Protospirura muricola*	*Trichostrongylidae*	*Trichuris mastomysi*	*Syphacia* sp.
	mean (sup.)	mean (sup.)	mean (sup.)	mean (sup.)	mean (sup.)
Hymenolepis sp.	0.09 (0.55)	0.58 (0.80)	0.31 (0.66)	0.20 (0.61)	-0.01 (0.49)
Davaineidae		0.15 (0.58)	0.06 (0.53)	0.08 (0.54)	0.16 (0.58)
Protospirura muricola			0.29 (0.65)	0.18 (0.60)	0.02 (0.51)
Trichostrongylidae				0.16 (0.58)	0.07 (0.54)
Trichuris mastomysi					0.14 (0.58)

Figure 2: Explanatory and predictive power of the presence-absence model based on the A) AUC and B) Tjur R² values, derived after five-fold cross-validation.

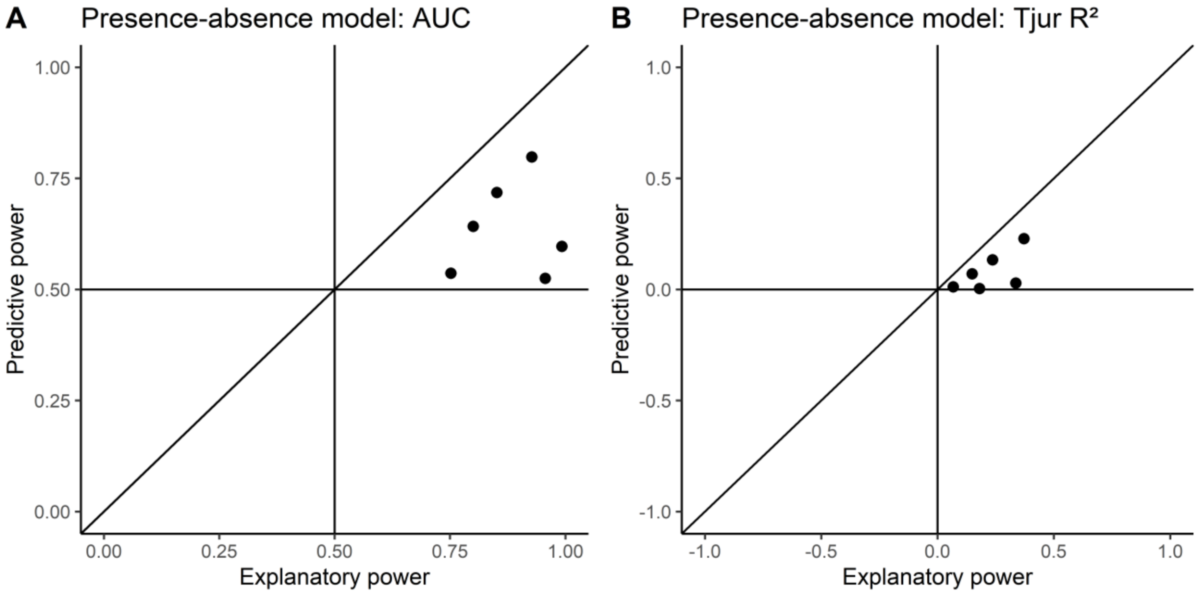

Figure 3: Explanatory and predictive power of the abundance COP model based on the R^2 values, derived after five-fold cross-validation.

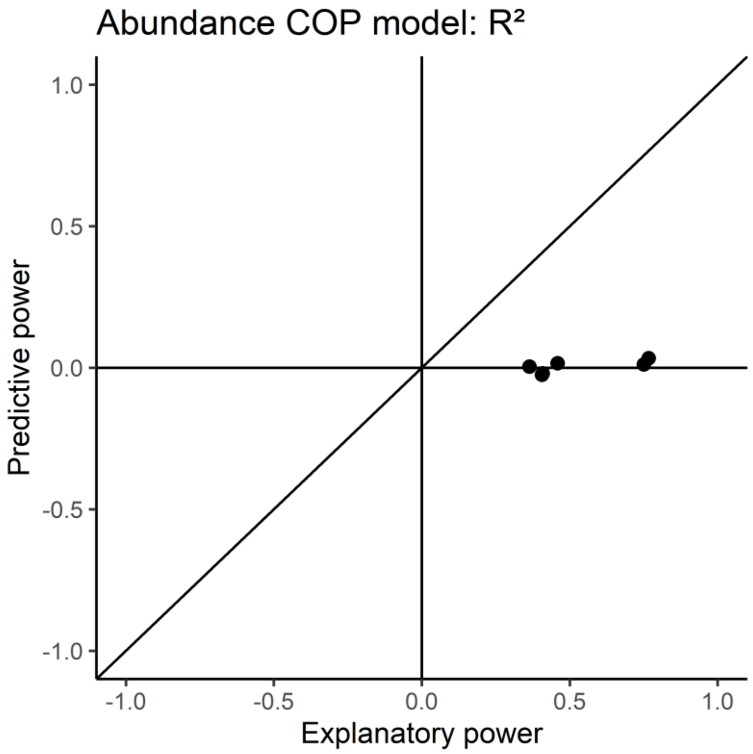

Figure 4: Differences in mean infection probability (± 95% credible interval) between adults and juveniles for (A) *Hymenolepis sp.*, (B) *Davaineidae*, (C) *Trichostrongylidae*, (D) *Protospirura muricola* and (E) *Trichuris mastomysi* derived from the presence-absence model.

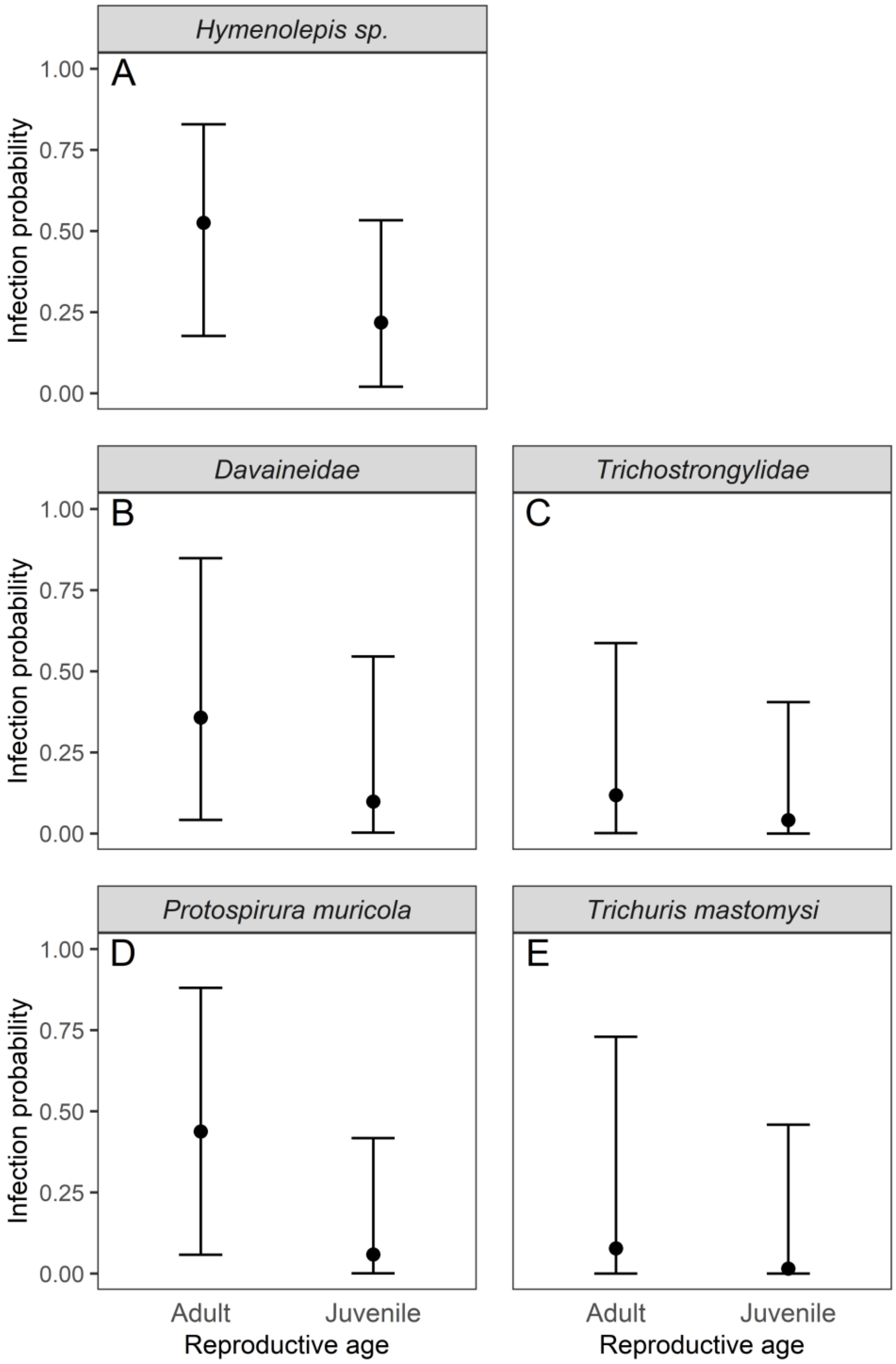

Figure 5: Predicted difference in mean log parasite load (± 95% credible interval) of *Davaineidae* between adults and juveniles, derived from the abundance COP model. Dots represent the observed individual parasite load.

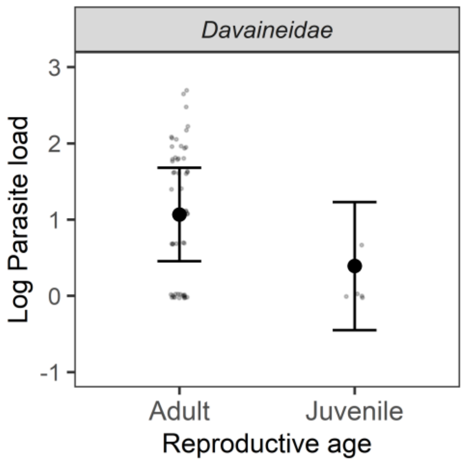

Figure 6: Differences in mean infection probability (± 95% credible interval) between males and females for (A) *Davaineidae* and (B) *Protospirura muricola* derived from the presence-absence model.

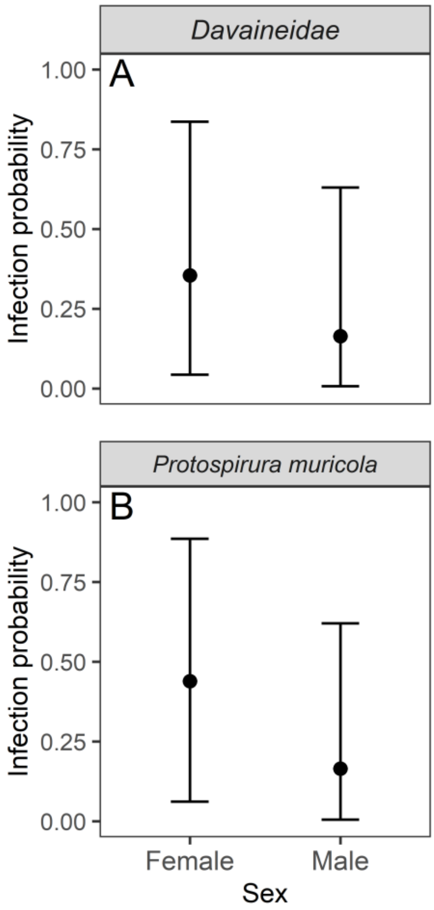

Figure 7: Predicted differences in mean log parasite load (± 95% credible interval) of (A) *Hymenolepis sp.* and (B) *Protospirura muricola* between males and females, derived from the abundance COP model. Dots represent the observed individual parasite load.

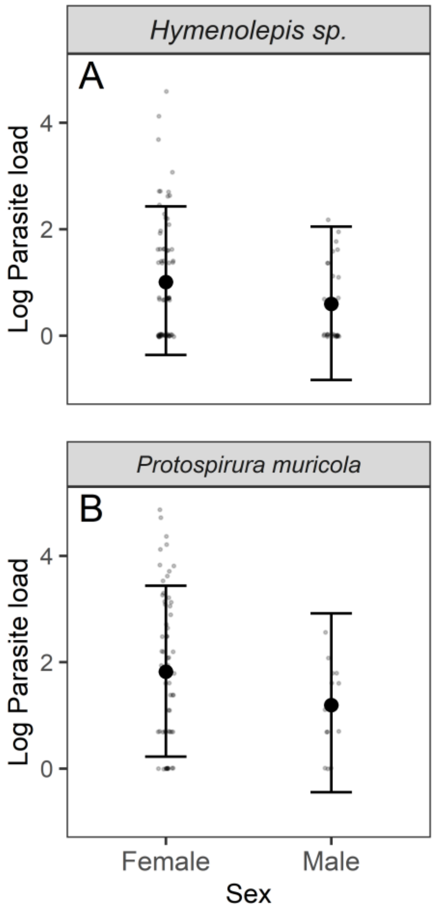

Figure 8: Predicted correlation between exploration behavior and infection probability of (A) *Davaineidae*, (B) *Protospirura muricola* and (C) *Trichuris mastomysi* derived from the presence-absence model. The grey bar represent the 95% credible interval for the predicted correlation and the dots are the observed values for each individual.

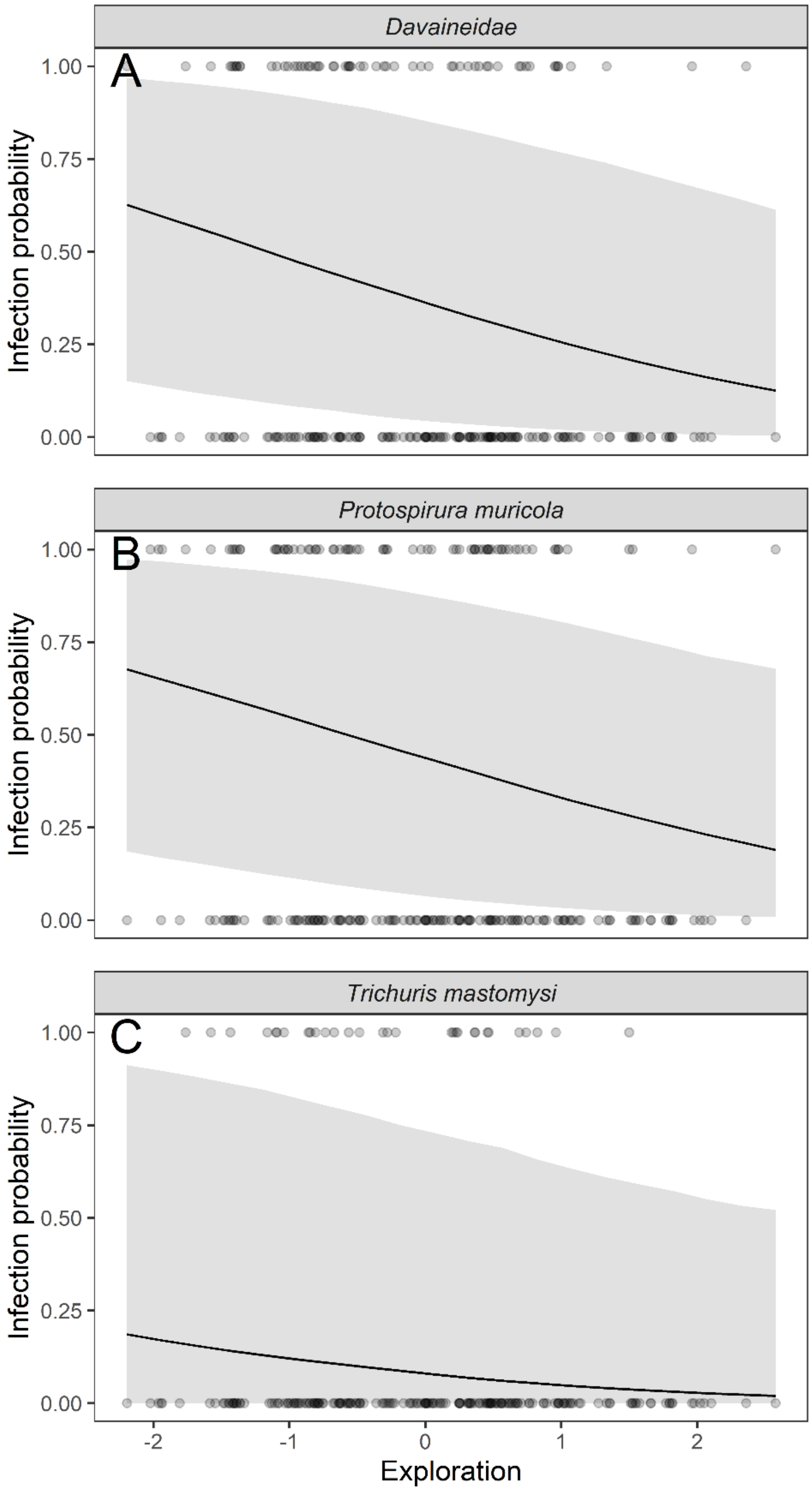

Figure 9: Predicted correlation between the individuals exploration behavior and log parasite load of *Trichostrongulidae*. Grey bar represents the 95% credible interval around the prediction and the dots represent the actual observed parasite load per individual.

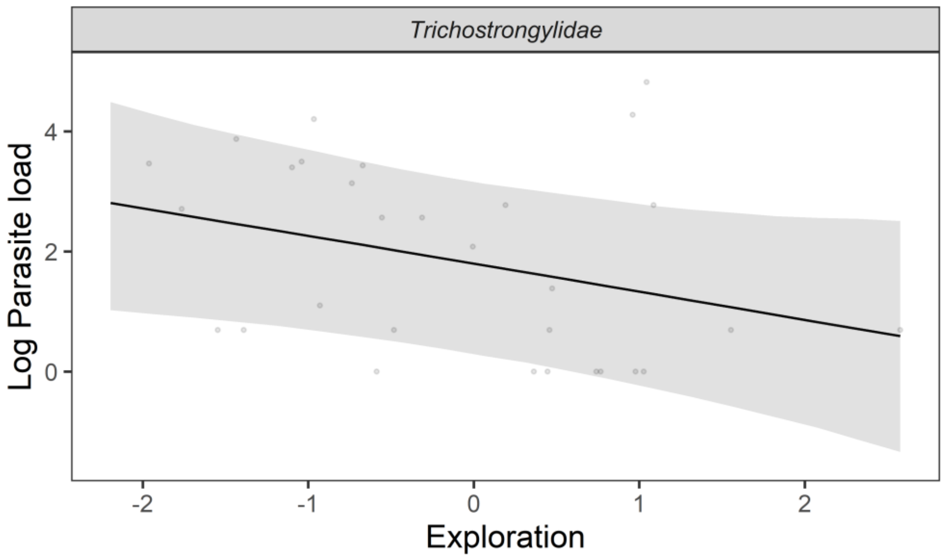

Figure 10: Predicted correlation between stress-sensitivity and infection probability of *Davaineidae* derived from the presence-absence model. The grey bar represent the 95% credible interval for the predicted correlation and the dots are the observed values for each individual.

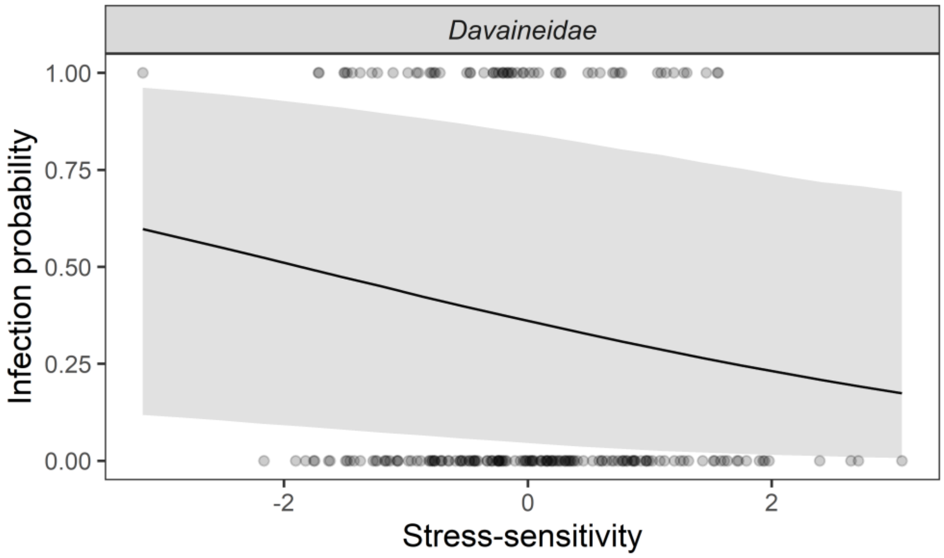

Figure 11: Predicted differences in mean infection probability (± 95% credible interval) of *Protospirura muricola* between individuals with (positive) and without (negative) antibodies against the Morogoro virus, derived from the presence-absence model.

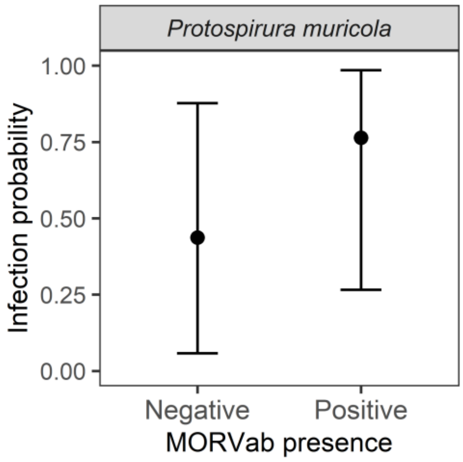

Supplement: Supplementary file 1 [file Data_Sheet_1.pdf]
